# Comparison of argon plasma coagulation in management of upper gastrointestinal angiodysplasia and gastric antral vascular ectasia hemorrhage

**DOI:** 10.1186/1471-230X-12-67

**Published:** 2012-06-09

**Authors:** Yi-Chun Chiu, Lung-Sheng Lu, Keng-Liang Wu, William Tam, Ming-Luen Hu, Wei-Chen Tai, King-Wah Chiu, Seng-Kee Chuah

**Affiliations:** 1 Division of Hepato-Gastroenterology, Department of Internal Medicine, Kaohsiung Chang Gung Memorial Hospital and Chang Gung University College of Medicine, 123, Ta-Pei Road, Niao-Sung District, Kaohsiung City, 833, Taiwan; 2 Department of Gastroenterology and Hepatology, Royal Adelaide Hospital, Adelaide, South Australia

**Keywords:** Endoscopic argon plasma coagulation, Angiodysplasia, Gastric antral vascular ectasia

## Abstract

**Background:**

Vascular ectasias, including gastric antral vascular ectasia (GAVE) and angiodysplasia, are increasingly recognized as important sources of gastrointestinal bleeding. This study investigated and compared the efficacies and outcomes of treatment of upper gastrointestinal (UGI) angiodysplasia and GAVE hemorrhage by endoscopic argon plasma coagulation (APC).

**Methods:**

From January 2006 to December 2009, 46 patients diagnosed with upper GI bleeding caused by angiodysplasia or GAVE at a tertiary hospital were recruited into this study. They included 26 males and 20 females with an average age of 65.6 years (range, 45–90 years). All patients underwent APC for hemostasis during an endoscopic procedure. Parameters such as underlying co-morbidities, number of endoscopic treatment sessions, recurrent bleeding, and clinical outcomes during follow-up were analyzed.

**Results:**

The 46 patients with UGI vascular ectasia hemorrhage included 27 patients with angiodysplasia and 19 with GAVE. The patients with angiodysplasia were older than those with GAVE (71.6 ± 10.2 years versus 61.8 ± 11.9 years, *P* = 0.005). More GAVE patients than angiodysplasia patients had co-existing liver cirrhosis (63.2% versus 25.9%, *P* = 0.012). The patients with GAVE had a higher rate of recurrent bleeding (78.9% versus 7.4%, *P* < 0.001) and required more treatment sessions to achieve complete hemostasis (2.4 ± 1.4 versus 1.1 ± 0.1, *P* < 0.001) than those with angiodysplasia. Univariate analysis demonstrated that age greater than 60 years (odds ratio (OR) = 8.929, *P* = 0.003), GAVE (OR = 0.021, *P* < 0.001), and previous radiation therapy (OR = 11.667, *P* = 0.032) were associated with higher rates of recurrent bleeding. Further multivariate analysis revealed that GAVE was the only independent risk factor for recurrent bleeding after APC treatment (OR = 0.027, *P* < 0.001).

**Conclusion:**

Endoscopic hemostasis with APC is a safe treatment modality for both angiodysplasia and vascular ectasia bleeding. The efficacy of APC treatment is greater for angiodysplasia than for vascular ectasia bleeding. GAVE patients have a higher recurrent bleeding rate and may require multiple treatment sessions for sustained hemostasis.

## Background

Gastrointestinal vascular ectasia comprises angiodysplasia, gastric antral vascular ectasia (GAVE), and other forms of telangiectasia related to multisystemic disease, such as hereditary hemorrhagic telangiectasia, but not vascular tumors and Dieulafoy’s lesion [1,2]. Angiodysplasias are typically discrete, flat or slightly raised, bright-red lesions 2 to 10 mm in size. GAVE is also called “watermelon stomach” due to the characteristic endoscopic findings of linear, friable, red streaks radiating from the pylorus. GAVE and angiodysplasia are the most common forms of upper gastrointestinal (UGI) vascular ectasia. Both are increasingly recognized as important sources of GI bleeding, accounting for up to 4% and 3% of cases of upper and lower GI bleeding, respectively [1-4]. The clinical presentation ranges from chronic gastrointestinal blood loss that leads to chronic anemia to serious gastrointestinal events such as melena or hematemesis, which occur especially in patients with underlying conditions leading to bleeding tendencies, such as liver cirrhosis, or uremia.

Various thermal modalities are used to treat GI vascular ectasia, including Neodymium: Yttrium Aluminum Garnet (Nd: YAG) laser, multipolar electrocoagulation, and argon plasma coagulation (APC). APC was reported to be as effective as laser photocoagulation and multipolar electrocoagulation and to have advantages over other non-contact treatments for use in difficult-to-access areas [5]. However, few studies have evaluated the outcomes and prognoses of patients with upper GI vascular ectasia hemorrhage treated with APC. There is also little information on the efficacy of APC at treating different types of UGI vascular ectasia hemorrhage. In the current study, we aimed to investigate and compare the efficacies of endoscopic APC treatment of patients with different forms of UGI vascular ectasia hemorrhage and their treatment outcomes.

## Methods

From January 2006 to December 2009, we retrospectively reviewed the medical records of 46 consecutive patients diagnosed with upper GI bleeding caused by angiodysplasia or GAVE at a university-affiliated tertiary care center. The patients included 26 males and 20 females with an average age of 65.6 years (range, 45–90 years). All patients underwent APC for hemostasis during an endoscopic procedure. UGI bleeding was diagnosed by (1) clinical signs, such as hematemesis, coffee ground vomitus, hematochezia, or melena or (2) endoscopic visualization of active bleeding, adherent blood clots, or coffee-ground material in the stomach. Patients with (1) possible bleeding lesions other than vascular ectasia visualized by endoscopy, (2) treatment by hemostatic modalities other than APC, or (3) an inability to give informed consent were excluded. The study received approval (No. 99-3027B) from the ethics committee of our institution and conformed to its guidelines.

### Endoscopic treatment

Unless contraindicated, intramuscular hyoscine butylbromide (20 mg) was administered as an antispasmodic agent approximately 5 minutes before the start of the procedure. With consent from each patient, APC was performed through the working channel of the endoscope under direct visualization by using an electrosurgical generator (PSD 60; Olympus, Tokyo, Japan) and a 2.3-mm probe (argon gas flow, 1.0–2.0 L/min; power 40 W). All lesions were treated until the mucosa became white in appearance after electrocoagulation. The procedures were repeated for upper GI vascular ectasias if there was recurrent GI bleeding (as evidenced by hematemesis or melena) or a need for further transfusion. Rebleeding was defined as a new onset of hematemesis, coffee-ground vomitus, hematochezia, or melena after 24 h of stable vital signs and hematocrit following endoscopic treatment. After endoscopic therapy, patients were treated with a proton pump inhibitor as follows: an 80-mg pantoprazole bolus was administered intravenously, followed by intravenous pantoprazole at 80 mg per day until alimentation was possible and oral pantoprazole at 40 mg per day thereafter. The patient data collected included underlying co-morbidities, pre-treatment hemoglobin, platelet count, prothrombin and activated partial thrombin times, endoscopic findings, number of endoscopic treatment sessions, evidence of recurrent bleeding, and clinical outcome(s) during follow-up.

### Statistical analysis

Continuous variables are expressed as the mean and standard deviation (SD). The continuous variables were analyzed using the Mann–Whitney U test. Categorical variables are expressed as totals and as percentages and were analyzed using the chi-square test or Fisher’s exact test. Univariate and multivariate logistic regression were used to analyze the factors related to recurrent bleeding after APC treatment. A *P* value less than 0.05 was considered statistically significant. All statistical analyses were performed using SPSS 17.0 (SPSS, Inc., Chicago, IL, USA).

## Results

The diagnosis was angiodysplasia in 27 patients and GAVE in 19. Seven (25.9%) patients with angiodysplasia had liver cirrhosis, 6 (22.2%) suffered from end-stage renal disease, and 1 (3.7%) had hereditary hemorrhagic telangiectasia. Five of 7 cirrhotic patients had hepatoma; 2 were treated by transarterial embolization via the hepatic artery, 1 by radiation therapy, and 2 by supportive therapy. Twenty-two of the patients with angiodysplasia had lesions located in the stomach, including 2 with lesions in the fundus, 10 with lesions in the body, 7 with lesions in the antrum, and 3 with lesions at multiple sites, while the remaining 5 patients had lesions in the duodenum. Concomitant gastric and colonic angiodysplasia was found in 1 patient, and both sites were successfully treated with APC. Of the 19 GAVE patients, 12 (63.2%) had liver cirrhosis, while only 2 had end-stage renal diseases. Seven (36.8%) patients had hepatomas; 6 were treated by transarterial embolization via the hepatic artery and 5 by radiation therapy. All of these 5 patients received external beam radiation therapy after transarterial embolization. Concurrent portal hypertensive gastropathy (PHG) was observed in 5 GAVE patients. During endoscopic examination, active bleeding was found in 70.4% of patients with angiodysplasia and 89.5% of GAVE patients. The pattern of active bleeding in all of these cases was oozing. The clinical characteristics, blood test data endoscopic findings, and co-existing diseases of the 2 disease groups are shown in Table 1. None of our patients was using anti-platelet or anti-inflammatory drugs, but 1 of the patients with angiodysplasia had a history of using aspirin. However, there was no rebleeding observed after APC treatment in this patient. The patients with angiodysplasia were older than those with GAVE (71.6 ± 10.2 years versus 61.8 ± 11.9 years, *P =* 0.005). A greater proportion of the GAVE patients than the angiodysplasia patients had co-existing liver cirrhosis (63.2% versus 25.9%, *P* = 0.012). Greater proportions of the GAVE patients than the angiodysplasia patients had histories of previous transarterial embolization (31.6% versus 7.4%, *P* = 0.051) and radiation therapy (26.3% versus 3.7%, *P* = 0.068), although neither of these differences reached significance.

**Table 1:** Clinical characteristics, blood-test data, and co-existing diseases in patients with angiodysplasia and gastric antral vascular ectasia (GAVE)

	Angiodysplasia N = 27	GAVE N = 19	*P* value
Age, years, mean ± SD*	71.6 ± 10.2	61.8 ± 11.9	0.005
Gender, M/F	15/12	11/8	0.875
Hemoglobin level (g/L), mean ± SD*	84.0 ± 32.0	82.0 ± 21.0	0.971
Platelet count (×10^9^/L), mean ± SD*	162.3 ± 84.2	142.1 ± 111.4	0.192
Prothrombin time (sec), mean ± SD*	11.1 ± 1.5	11.1 ± 3.3	0.551
Activated partial thromboplastin time (sec), mean ± SD*	30.1 ± 5.1	27.0 ± 2.8	0.095
Active blood oozing on endoscopy, n (%)	19 (70.4)	17 (89.5)	0.160
Liver cirrhosis, n (%)	7 (25.9)	12 (63.2)	0.012
End-stage renal disease, n (%)	6 (22.2)	2 (10.5)	0.440
Hepatoma, n (%)	5 (18.5)	7 (36.8)	0.19
§Transarterial embolization, n (%)	2 (7.4)	6 (31.6)	0.051
§Radiation therapy, n (%)	1 (3.7)	5 (26.3)	0.068

* SD: 1 standard deviation

§ as previous treatment for hepatoma

Initial hemostasis was achieved by APC during endoscopy in all cases. Recurrent bleeding occurred in 36.9% of these patients (17/46), including 7.4% (2/27) of those with angiodysplasia and 78.9% (15/19) of those with GAVE; the rebleeding was from the previous treatment site in all cases. The median duration of rebleeding after APC treatment was 14 days (7 to 21 days) in patients with angiodysplasia and 23 days (7 to 116 days) in those with GAVE. There was no complication related to endoscopic treatment in patients with either condition. There was also no mortality related to GI bleeding in the angiodysplasia patients, but 3 GAVE patients died of recurrent GI bleeding. All of these 3 patients suffered from co-morbidities such as concurrent liver cirrhosis and hepatoma, sepsis, or respiratory failure. As shown in Table 2, APC was more effective at achieving complete hemostasis and hospital discharge in patients with angiodysplasia than in those with GAVE (100% versus. 78.9%, *P* = 0.024). Patients with GAVE had a higher rate of rebleeding (78.9% versus 7.4%, *P* < 0.001) and required a greater number of treatment sessions to achieve hemostasis (2.4 ± 1.4 versus 1.1 ± 0.1, *P* < 0.001) than those with angiodysplasia. Bleeding-related mortality was also higher, although not significantly so, in GAVE patients than in those with angiodysplasia (15.8% versus 0%, *P* = 0.064). The presence of active oozing was not significantly associated with a higher rate of mortality (8.3% versus 0%, *P* = 1.0) or rebleeding (41.7% versus 20%, *P* = 0.282) after APC treatment. The results of univariate and multivariate analyses of recurrent bleeding using a logistic regression model are shown in Table 3. Univariate analysis demonstrated that age greater than 60 years (odds ratio (OR) = 8.929, *P =* 0.003), GAVE (OR = 0.021, *P* < 0.001), and previous radiation therapy (OR = 11.667, *P* = 0.032) were associated with higher rates of recurrent bleeding. Further multivariate analysis revealed that only GAVE was an independent risk factor for recurrent bleeding after APC treatment (OR = 0.027, *P* < 0.001).

**Table 2:** Comparison of the responses of angiodysplasia and GAVE to treatment with endoscopic argon plasma coagulation

	Angiodysplasia N = 27	GAVE N = 19	*P* value
Treatment sessions, mean ± SD*	1.1 ± 0.1	2.4 ± 1.4	<0.001
Recurrent bleeding, n (%)	2 (7.4)	15 (78.9)	<0.001
Complete hemostasis and hospital discharge, n (%)	27 (100%)	15 (78.9)	0.024
Mortality related to bleeding, n (%)	0 (0)	3 (15.8)	0.064

* SD: 1 standard deviation

**Table 3:** Univariate and multivariate analyses of the associations of individual parameters with recurrent bleeding in patients with bleeding vascular ectasia

Parameter*	Univariate			Multivariate		
	OR	95% CI	*P*	OR	95% CI	*P*
Age	8.929	2.135–37.337	0.003	6.796	0.564–70.649	0.109
Gender	1.259	0.377–4.204	0.708	_	_	_
Type of vascular ectasia	0.021	0.003–0.131	<0.001	0.027	0.003–0.247	<0.001
Acute blood oozing	2.857	0.529–15.384	0.222	2.639	0.087–80.114	0.557
Liver cirrhosis	3.175	0.913–11.033	0.069	1.562	0.118–20.610	0.775
ESRD	1.029	0.213–4.973	0.972	_	_	_
Hepatoma	2.091	0.547–7.989	0.281	2.214	0.023–211.953	0.733
Transarterial embolization	3.611	0.739–17.644	0.113	1.602	0.007–361.954	0.865
Radiation therapy	11.667	1.228–110.803	0.032	11.627	0.276–500	0.198

* Cut-offs: Age: ≥60 or <60 years, gender: male or female, type of vascular ectasia: angiodysplasia or GAVE, active blood oozing on endoscopy: yes or no, liver cirrhosis: yes or no, ESRD (end-stage renal disease): yes or no, hepatoma: yes or no, previous transarterial embolization: yes or no

## Discussion

Angiodysplasia in the GI tract is a vascular lesion the actual cause of which remains unknown. Many hypotheses have been advanced in the literature, but most of these explanations are obscure. GI angiodysplasia has been reported to be associated with other systemic diseases, such as aortic valvular diseases, systemic sclerosis, hereditary hemorrhagic telangiectasis, and end-stage renal disease [6,7]. Some have speculated that it may be the result of chronic low-grade venous obstruction secondary to increased intramural pressure [8] or local mucosal hypoxia [9]. Markwick and colleagues [6] reported that the lesions were mostly multiple and located in the proximal part of the stomach, while Weaver and colleagues [10] found angiodysplasia most often located at the junction of the middle to the distal third of the stomach, similarly to the current study. Likewise, the pathogenesis of GAVE is also unknown but appears to be related to liver cirrhosis in most cases. Other disorders are also associated with GAVE, including chronic renal failure [11], chronic valvular, ischemic, or hypertensive heart disease [12], and a variety of autoimmune diseases [13]. The endoscopic appearance of GAVE is unlike that of angiodysplasia, and Novitsky et al. described 4 different patterns [7]. The most common of these is antral disease with classic raised convoluted ridges covered by ectatic vascular tissue radiating out from the pylorus. Other features include radiating flat stripes, scattered multiple mucosal lesions, and lesions of mixed type. Generally, linear lesions within the antrum are observed in non-cirrhotic patients and diffuse lesions in cirrhotic patients [14,15]. Regardless of the pattern, bleeding of GAVE lesions begins with injury by either gastric acid or intraluminal food to the mucosal epithelium overlying the engorged vessels. The severity of bleeding ranges from minor, but chronic upper GI blood loss to severe melena and hematemesis that leads to anemia and requires blood transfusion. In our case series, the patients with angiodysplasia were older than those with GAVE, although both groups averaged over 60 years of age and were composed of elderly individuals. The younger age of GAVE patients may be related to the higher prevalence of liver cirrhosis in this group, which in turn can be explained by the high prevalence rates of Hepatitis B (10–15%) and Hepatitis C (1–3%) infection in Taiwan [16-19]. Cirrhosis of the liver was found in 30% to 70% of patients with GAVE [7,20,21]. Therefore, PHG is usually present concomitantly with and requires careful differentiation from vascular ectasia. In contrast to GAVE, which usually involves the gastric antrum, PHG generally affects the fundus and corpus of the stomach. However, GAVE and PHG are considered to be separate clinical entities because of the frequent presence of GAVE in the absence of portal hypertension [21]. PHG is rarely found as the sole cause of significant UGI bleeding in patients with portal hypertension [22]. Twelve of the GAVE patients in the current study had liver cirrhosis. Of these, 5 had concomitant PHG, and none was found to be bleeding from PHG during endoscopic examination.

Many endoscopic techniques are used to treat UGI vascular ectasias, including sclerotherapy, multipolar electrocoagulation, argon and laser photocoagulation, and APC [1]. Early publications used mostly Nd: YAG laser coagulation, which produced acceptable results but was relatively expensive [23-26]. In the last decade, APC has been proven to be at least as effective. [5] In published series of treatment of GAVE bleeding; the number of sessions required to achieve hemostasis was lower when APC was used than when laser or bipolar techniques were used [25-27]. The current study found that the use of APC to treat both UGI vascular ectasia and GAVE attained results comparable to those of other studies, with success rates of near 80% for GAVE [28,29]and 100% for UGI angiodysplasia [30,31].

APC treatment is characterized by noncontact coagulation, which allows tangential application and thus treatment of the target site in a uniform manner to a depth of approximately 1 to 3 mm, which is sufficient to coagulate the superficial blood vessels [32]. Successful APC therapy leads to whitish coagulation of the mucosa and the disappearance of the underlying vascular structures. The coagulation depth of APC depends on the power generator setting, the distance from the target tissue, and the duration of the application [33]. Histologically, the tortuous ectatic vessels of vascular ectasia extend superficially over the submucosal layer [34]. For this reason, variable power settings (30–100 W) and flow rates of argon gas (0.8–2 liter/min) have been reported to be safe and effective for hemostasis of bleeding vascular ectasia [1,5,27-29,32]. To our knowledge, there are no publications comparing various power settings and flow rates for safety and efficacy for this application. An experienced gastroenterologist can adjust the distance between the APC probe and the target lesion and the duration of application to achieve a satisfactory effect at a variety of settings. Therefore, the operator’s experience and technique are quite crucial to successful APC treatment.

We observed that the patients with GAVE had a higher rebleeding rate than those with angiodysplasia despite no significant differences in platelet count, prothrombin time, activated partial thromboplastin time, or rates of end-stage renal disease and hepatoma between these 2 groups. On one hand, the prevalence of liver cirrhosis (62.3%) in the GAVE patients of the current study, while consistent with the reported prevalence rates of 30% to 70% in published series [7,20,27,28], was higher than in the angiodysplasia patients. The bleeding tendency associated with liver cirrhosis may have been responsible for the higher rebleeding rate, and even the higher mortality rate, in the GAVE group. On the other hand, the endoscopic appearance of angiodysplasia is single or multiple discrete red lesions 2 to 10 mm in size, whereas GAVE is characterized chiefly by erythematous stripes radiating in a spoke-like fashion from the pylorus to the antrum, mimicking a “watermelon” appearance. Therefore, the area with potential for bleeding is obviously larger in GAVE patients than in those with angiodysplasia. The higher rebleeding rate in the GAVE patients despite careful and thorough treatment by APC is a therefore a predictable outcome. However, despite the relatively small size of the lesions, the rebleeding rate of angiodysplasia after endoscopic treatment is not inconsiderable in some reports; [33] it may also be higher in uremic patients [32,33,35,36]. Therefore, this report may have underestimated the rebleeding rate of angiodysplasia lesions because of the small number of cases.

Both the Vienna and Asia-Pacific consensuses recommended intravenous high-dose proton-pump inhibitor (PPI) therapy after successful endoscopic hemostasis of high-risk bleeding ulcers [37,38]. Nevertheless, many studies have shown that high-dose PPI treatment does not further reduce the rate of rebleeding compared with non-high-dose PPI treatment [39-41]. The optimal dosing of PPIs in these patients remains controversial. However, maintaining an intragastric pH of >6.0 allows stabilization of the clot, which stops peptic ulcer bleeding and prevents rebleeding [42-44]. In the case of vascular ectasia bleeding, the use of PPIs could prevent rebleeding after APC hemostasis and enhance procedure-induced ulcer healing. In this study, we used non-high-dose PPI treatment and observed an overall rebleeding rate of 36.9%, including 7.4% of the angiodysplasia patients and 78.9% of the GAVE patients. However, the evidence on the optimal dosing of PPIs following APC treatment of bleeding vascular ectasia is limited, and no study gave detailed information on the dose of PPIs prescribed [27-29]. Therefore, there is need for further study to clarify this issue.

There was no complication related to APC during endoscopy or after treatment in the 74 treatment sessions of 46 patients of the present study. APC is a non-contact thermal method of hemostasis that uses argon plasma to transfer electrical energy to the target tissue. Its characteristics include rapid treatment of multiple or extensive lesions and decreased depth of penetration. However, all of the complications that have been reported with other thermal hemostasis techniques, such as submucosal emphysema, superficial ulceration, fibrotic contracture, stricture, and perforation, can still occur [45]. The perforation rate has been reported to range from 0% to 8% and occurs most frequently when APC is used for ablation of Barrett’s esophagus and palliative ablation for malignant stenosis [32,45]. The depth of energy penetration is crucial to the occurrence of perforation after thermal hemostasis. The relatively superficial coagulation required for treatment of UGI vascular ectasia should largely avoid this serious complication, making non-contact APC a safe and effective option

Three GAVE patients in the current study died of recurrent GI bleeding, and all of them suffered from other co-morbidities, such as concurrent liver cirrhosis and hepatoma, sepsis, or respiratory failure. Surgical intervention, mainly antrectomy, has been reported for treatment of recurrent bleeding of GAVE lesions [21]. However, surgery is usually reserved for patients who fail endoscopic therapies and, moreover, are often elderly with major co-morbid illnesses. The post-operative morbidity in these patients was reported to be up to 23% with a mortality rate of about 2.5% [46,47]. Therefore, in consideration of the terminal disease status of these 3 patients, surgery was not considered.

## Conclusion

Endoscopic hemostasis with APC is a safe modality for treatment of both angiodysplasia and vascular ectasia bleeding. APC is more effective at treating angiodysplasia than at treating vascular ectasia bleeding. GAVE is associated with a higher rebleeding rate and may require multiple sessions to achieve sustained hemostasis.

## Abbreviations

APC: Argon plasma coagulation; UGI: Upper gastrointestinal; GAVE: Gastric antral vascular ectasia; PHG: Portal hypertensive gastropathy; PPI: Proton pump inhibitor.

## Competing interests

The authors declare that they have no competing interests.

## Authors’ contributions

Yi-Chun Chiu executed the study and drafted the manuscript. Lung-Sheng Lu, Keng-Liang Wu, Ming-Luen Hu, and Wei-Chen Tai collected the cases and performed clinical follow-up. William Tam revised and corrected the English of the manuscript. King-Wah Chiu critically revised the manuscript for important intellectual content. Seng-Kee Chuah (corresponding author) conceived and designed the study and gave final approval of the manuscript. All authors read and approved the final manuscript.

## Pre-publication history

The pre-publication history for this paper can be accessed here:

http://www.biomedcentral.com/1471-230X/12/67/prepub
